# The proneural transcription factor Atoh1 promotes odontogenic differentiation in human dental pulp stem cells (DPSCs)

**DOI:** 10.1186/s12860-025-00530-2

**Published:** 2025-01-20

**Authors:** Camila Sabatini, Huey-Jiun Lin, Galib Ovik, Richard Hall, Techung Lee

**Affiliations:** 1 Department of Restorative Dentistry, School of Dental Medicine, University at Buffalo, 3435 Main Street, Buffalo, NY 14214 USA; 2 Department of Oral Surgery, University at Buffalo, 3435 Main Street, Buffalo, NY 14214 USA; 3 Department of Biochemistry, University at Buffalo, 3435 Main Street, Buffalo, NY 14214 USA

**Keywords:** Atoh1, Transcription factor, Dental pulp stem cells, Odontogenesis

## Abstract

**Background:**

Bioengineering of human teeth for replacement is an appealing regenerative approach in the era of gene therapy. Developmentally regulated transcription factors hold promise in the quest because these transcriptional regulators constitute the gene regulatory networks driving cell fate determination. Atonal homolog 1 (Atoh1) is a transcription factor of the basic helix-loop-helix (bHLH) family essential for neurogenesis in the cerebellum, auditory hair cell differentiation, and intestinal stem cell specification. The functional versatility of Atoh1 prompted us to test the possibility that Atoh1 may intersect the dental pulp stem cell (DPSC) gene regulatory network governing odontogenic differentiation.

**Methods:**

We isolated DPSCs from human dental pulps and treated the cells with a replication-deficient adenoviral vector to achieve robust ectopic expression of Atoh1, following which the growth and odontogenic differentiation profiles of DPSCs were characterized.

**Results:**

DPSCs harboring the Atoh1 expression vector exhibited an approximately 3,000-fold increase in the expression of Atoh1 compared to the negative control, leading to increased DPSC proliferation in the growth medium (*P* < 0.05). In the odontogenic medium, Atoh1 caused an early induction of BMP2 (*P* < 0.001) followed by a late induction of BMP7 (*P* < 0.01) and increased Wnt signaling (*P* < 0.01). The increased BMP/Wnt signaling led to up to 8-fold increased expression of the master osteogenic transcription factor Osterix (*P* < 0.005) while exhibiting no significant effect on Runx2 or Dlx5, which are abundantly expressed in DPSCs. Atoh1 stimulated expression of type I collagen (*P* < 0.005) and small integrin-binding ligand, N-linked glycoproteins (SIBLINGs) such as bone sialoprotein (*P* < 0.001), dentin matrix protein 1 (*P* < 0.05), dentin sialophosphoprotein (*P* < 0.005), and osteopontin (*P* < 0.001), resulting in increased dentin matrix mineralization (*P* < 0.05). The odontogenic phenotype is associated with metabolic remodeling marked by enhanced glycolytic flux and attenuated mitochondrial metabolic enzyme activities.

**Conclusions:**

Atoh1, despite being a proneural transcription factor in development, possesses a novel odontogenic function upon ectopic expression in DPSCs. This in vitro study demonstrates a novel odontogenic mechanism mediated by ectopic expression of the transcription factor Atoh1 in human DPSCs. The finding may offer an innovative strategy for gene-based regeneration of the pulp-dentin complex.

**Supplementary Information:**

The online version contains supplementary material available at 10.1186/s12860-025-00530-2.

## Background

Tooth development and repair require coordinated extrinsic cues serving to guide the gene regulatory networks through induction of spatially and temporally regulated transcription factors [1]. Among the signaling cues regulating vertebrate tooth development are bone morphogenetic proteins (BMP), Wnt ligands, fibroblast growth factors (FGF), and Sonic Hedgehog (Shh), which activate an array of transcription factors such as Smad, β-catenin, Osterix, Runx2, and GLI1 [2–4]. These transcription factors govern stem cell function and tissue renewal through differential DNA binding and protein interaction, thus offering unique opportunities for dental tissue engineering and repair. Bioengineering of human teeth for replacement may become feasible in the era of gene therapy. Developmentally regulated transcription factors hold great promise toward the aim because these proteins constitute the gene regulatory networks driving cell fate determination through differential interaction with the gene promoters and regulation of the chromatin remodeling process.

Atonal homolog 1 (Atoh1) is a DNA-binding protein that belongs to the basic helix-loop-helix (bHLH) family of dimeric transcription factors involved in the regulation of embryonic development, especially neurogenesis in the cerebellum and neurosensory development in the ear [5, 6]. Expression of Atoh1 has been found to be sufficient to drive auditory hair cell differentiation, prompting several clinical trials of Atoh1 gene therapy for hearing loss [7, 8]. The bHLH protein domain of Atoh1 binds to the promoter DNA motif designated as E-box and confers specificity on interaction with many transcriptional regulators, allowing Atoh1 to potentially regulate more than 600 diverse gene targets in various cells and tissues in a spatiotemporal manner [9]. Indeed, dysregulation of the Atoh1-mediated gene regulatory network can cause cerebellar neoplasia leading to medulloblastoma. Aside from its prominent roles in cochlear development and auditory hair cell regeneration, Atoh1 is also known to regulate intestinal stem cell differentiation and goblet cell function, which is a testament to its molecular contrivance and functional versatility [10].

A prominent feature of the Atoh1-mediated transcriptional network is that it is capable of inducing expression of physiologically diverse genes in a developmental stage- and tissue context-dependent manner. Notably, Atoh1 is known to participate in the signaling pathways mediated by BMP, Wnt, and Shh [11–13]. Since members of the BMP gene family and canonical Wnt signaling are functionally involved in promoting the osteogenic and odontogenic differentiation pathways, we hypothesize that Atoh1 may intersect the dental pulp stem cell (DPSC) gene regulatory network governing odontogenesis and regeneration of the pulp-dentin complex. Using an adenoviral vector to achieve robust ectopic expression of Atoh1 in human DPSCs, we demonstrate for the first time that Atoh1, despite being dubbed as a proneural transcription factor, possesses a novel pro-odontogenic property. We further demonstrate that this pro-odontogenic activity of Atoh1 is mediated at least in part by increased BMP and Wnt signaling. The finding may pave the way for future gene-based bioengineering of human teeth for regenerative tooth repair.

## Methods

### Tooth extraction

The protocol for collection of non-carious third molars from patients of the University at Buffalo School of Dental Medicine clinics was approved by the Institutional Review Board (STUDY00008199). The extracted tooth was disinfected in 70% ethanol for 1 min and briefly rinsed in normal saline, following which the tooth was cut open with a saw under a sterile condition for pulp tissue retrieval.

### Culture of human dental pulp stem cells (DPSCs)

DPSC isolation was described previously [14]. In brief, the pulp tissue was incubated in 5 mg/ml collagenase at 37 °C for 90 min with occasional agitation, following which tissue was gently triturated for 3 min. Cell suspension was collected, pelleted, and resuspended in a growth medium consisting of Advanced DMEM (Thermo Fisher Scientific, USA) supplemented with 10% fetal bovine serum (FBS), 2 mM GlutaMAX (Thermo Fisher Scientific), 50 µg/ml gentamycin, and 0.125 µg/ml amphotericin B. Cells were plated on a 60-mm dish maintained in a humidified 37 °C CO_2_ incubator for 2 days. Non-adherent cells were removed after 2 days, and adherent DPSCs were allowed to reach confluency prior to trypsinization with TrypLE (Thermo Fisher Scientific, USA). The use of Advanced DMEM, which contains antioxidants such as ascorbic acid phosphate and glutathione, allows DPSCs derived from young female donors (15–18 years of age) to grow for more than 20 trypsin passages without showing clear signs of cellular senescence. Data presented in the manuscript were collected from 3 to 13 passages.

### Induction of odontogenic differentiation

DPSCs plated on 24-well plates or 35-mm dishes were allowed to grow to ~ 90% confluency in the growth medium. To initiate odontogenic differentiation, the growth medium was replaced with the well-established osteogenic medium consisting of Advanced DMEM supplemented with 2% FBS, 2 mM Glutamax, 30 nM dexamethasone, 50 µM ascorbyl phosphate, and 10 mM β-glycerol phosphate. DPSCs were typically maintained in the odontogenic differentiation medium for up to 20 days with several changes of medium.

### Replication-deficient recombinant adenoviral vector

The use of the replication-deficient adenoviral vector (Adv) is approved by the Institutional Biosafety Committee (030BIO00000306). The viral vectors Adv-Null (no gene insert; negative control) and Adv-Atoh1 (Hath1) were kindly provided by Richard Salvi (University at Buffalo, Department of Communicative Disorders and Sciences). Viral amplification was performed in HEK293 cells to yield ∼10^9^ viral particles/ml as described previously [15]. DPSCs were treated with Adv-Null and Adv-Atoh1 in serum-free Advanced DMEM at a multiplicity of infection of ~ 100 for 2 h with occasional agitation, following which cells were washed twice with Hank’s Balanced Salt Solution (HBSS) and maintained in the growth or odontogenic medium as indicated in the figure legends.

### MTT cell proliferation assay

Cell proliferation was analyzed by MTT assay as described previously [14]. Briefly, DPSCs were plated on a 24-well plate with a cell density of 6,000 cells/well. For MTT dye conversion, cells were treated with 0.3 ml of the growth medium containing 0.125 mg/ml MTT at 37ºC for 20 min. The medium was then replaced with 0.2 ml of dimethyl sulfoxide (DMSO) for extraction of the formazan dye, following which the DMSO solution was transferred to a 96-well plate for optical measurement. The intensity of the formazan dye was measured by light absorbance at 540 nm.

### RNA isolation and qPCR quantification

The protocols were described previously [15]. In brief, total DPSC RNA was treated with DNase and extracted using an RNA isolation kit from Qiagen (USA) 4, 7, or 14 days after initiation of odontogenesis. cDNA synthesis was performed using iScript cDNA Synthesis kit (Bio-Rad, USA). cDNA was subsequently diluted 5–10-fold with dH_2_O and mixed with 20 µM primers and SYBR Green master mix (Bio-Rad, USA) for qPCR analysis. qPCR was performed using Azure Cielo 6 PCR instrument. β2 microglobulin (B2M) was used as the reference gene. Data were analyzed by the 2^∆∆Ct^ method. Oligonucleotides (Table 1) were purchased from OriGene Technologies (USA) or Thermo Fisher Scientific (USA).

**Table 1:** Oligonucleotides for qPCR

Gene	Forward primer	Reverse primer
B2M	TGCTGTCTCCATGTTTGATGTATCT	TCTCTGCTCCCCACCTCTAAGT
Atoh1	CCTTCCAGCAAACAGGTGAATGG	CTTTCCCACCTGCTTGCATT
BMP2	ATTCCCCGTGACCAGACTTT	CTTTCCCACCTGCTTGCATT
BMP7	CATGAGCTTCGTCAACCTCG	AAACCGGAACTCTCGATGGT
Osteocalcin	CGCTACCTGTATCAATGGCTGG	CTCCTGAAAGCCGATGTGGTCA
sFRP2	ATAAAAATGATGATGACAACGACATA	ATCTCCTTCACTTTTATTTTCAGTG
Axin2	CGATGAGTTTGCCTGTGGAG	CCGTCTCATCCTCCCAGATC
PDGF	CCAGCGACTCCTGGAGATAGA	CTTCTCGGGCACATGCTTAGT
VEGF-A	GCACCCATGGCAGAAGG	CTCGATTGGATGGCAGTAGCT
HIF1α	TATGAGCCAGAAGAACTTTTAGGC	CACCTCTTTTGGCAAGCATCCTG
Osterix	TTCTGCGGCAAGAGGTTCACTC	GTGTTTGCTCAGGTGGTCGCTT
Runx2	CCCAGTATGAGAGTAGGTGTCC	GGGTAAGACTGGTCATAGGACC
Dlx5	TACCCAGCCAAAGCTTATGCCG	GCCATTCACCATTCTCACCTCG
Collagen 1A1	GATTCCCTGGACCTAAAGGTGC	AGCCTCTCCATCTTTGCCAGCA
BSP	GGCAGTAGTGACTCATCCGAAG	GAAAGTGTGGTATTCTCAGCCTC
DMP1	GAGCAGTGAGTCATCAGAAGGC	GAGAAGCCACCAGCTAGCCTAT
DSPP	CAACCATAGAGAAAGCAAACGCG	TTTCTGTTGCCACTGCTGGGAC
OPN	CGAGGTGATAGTGTGGTTTATGG	GCACCATTCAACTCCTCGCTTTC
ALP	ACCATTCCCACGTCTTCACA	AGGGCTTCTTGTCTGTGTCA

### Alizarin red staining

Alizarin Red staining solution consists of 2% Alizarin Red dissolved in 50 mM pH4 sodium acetate buffer. DPSCs maintained in the odontogenic medium for 20 days were fixed in 4% paraformaldehyde at room temperature for 1 h, washed with dH_2_O three times, and immersed in Alizarin Red solution at room temperature with gentle agitation for 1 h. Stained cells were washed with dH_2_O three times, photographed, and extracted with 1% hydrochloric acid in 70% ethanol for 30 min. The extracts were quantified by light absorbance at 450 nm.

### Western blotting

The protocol for Western blotting was described previously [16]. In brief, cell lysates prepared in 1% SDS were mixed with SDS-PAGE sample buffer containing 1% β-mercaptoethanol and heat treated at 95ºC for 5 min. Proteins were fractionated by 10% SDS–PAGE, following which proteins were electrotransferred to Immobilon-P membrane. The membrane was briefly stained with 0.1% Ponceau S prepared in 1% acetic acid to verify equal protein loading. The Atoh1 antibody, purchased from Proteintech (USA; cat# 21215-1-AP), was diluted 1,000-fold for overnight membrane probing at 4ºC. The membrane was washed with TBST (20 mM Tris pH7.6, 150 mM NaCl, 0.05% Tween-20) and subsequently probed with 20 ng/ml secondary antibody conjugated to horse radish peroxidase (HRP) at room temperature for 1 h. Protein band signals were visualized with the SuperSignal chemiluminescent substrate (Pierce Biotechnology, USA) and digitally imaged using Bio-Rad (USA) ChemiDoc MP.

### Enzyme activity assays

Measurements of enzyme activities of glyceraldehyde-3-phosphate dehydrogenase (GAPDH), tartrate-resistant acid phosphatase (TRAP), α-ketoglutarate dehydrogenase complex (α-KGDC), and fatty acid oxidation (FAO) were set up in 96-well plates and assayed using colorimetric assay kits provided by Biomedical Research Service (University at Buffalo, New York, USA). Assays of GAPDH, α-KGDC, and FAO are based on reduction of the tetrazolium salt INT in a NADH-coupled enzymatic reaction to formazan after 1–2 h of incubation at 37ºC. The INT formazan exhibits an absorption maximum at 492 nm. TRAP assay is based on conversion of p-nitrophenol phosphate to nitrophenol in an acidic buffer at 37ºC, which exhibits an absorption maximum at 405 nm. Enzyme activities derived from optical density were each normalized by TRAP activity, which remain relatively unchanged during DPSC odontogenesis.

### Medium L-lactate assay

Medium L-lactate was quantified using an assay kit from Biomedical Research Service (New York, USA). The lactate assay is based on lactate dehydrogenase-mediated reduction of the tetrazolium salt INT. Spent medium samples were first deproteinized in 25% polyethylene glycol (PEG)-8000 on ice for 10 min. Supernatants were harvested and diluted with dH_2_O. Twenty µl of each deproteinized and diluted medium sample was mixed with 50 µl of Lactate Assay Solution in a 96-well plate and incubated at 37 °C for 60 min. Optical density was measured at 492 nm. Sample lactate concentrations were derived from a lactate standard curve performed at the same time.

### Statistical analysis

Comparisons of parameters between Adv-Null and Adv-Atoh1 are made with Student’s t-test (two-tailed). Data are expressed as mean ± standard deviation. A value of *P* < 0.05 is considered significant.

## Results

### Overexpression of Atoh1 promotes DPSC proliferation and odontogenesis

**Fig. 1:**
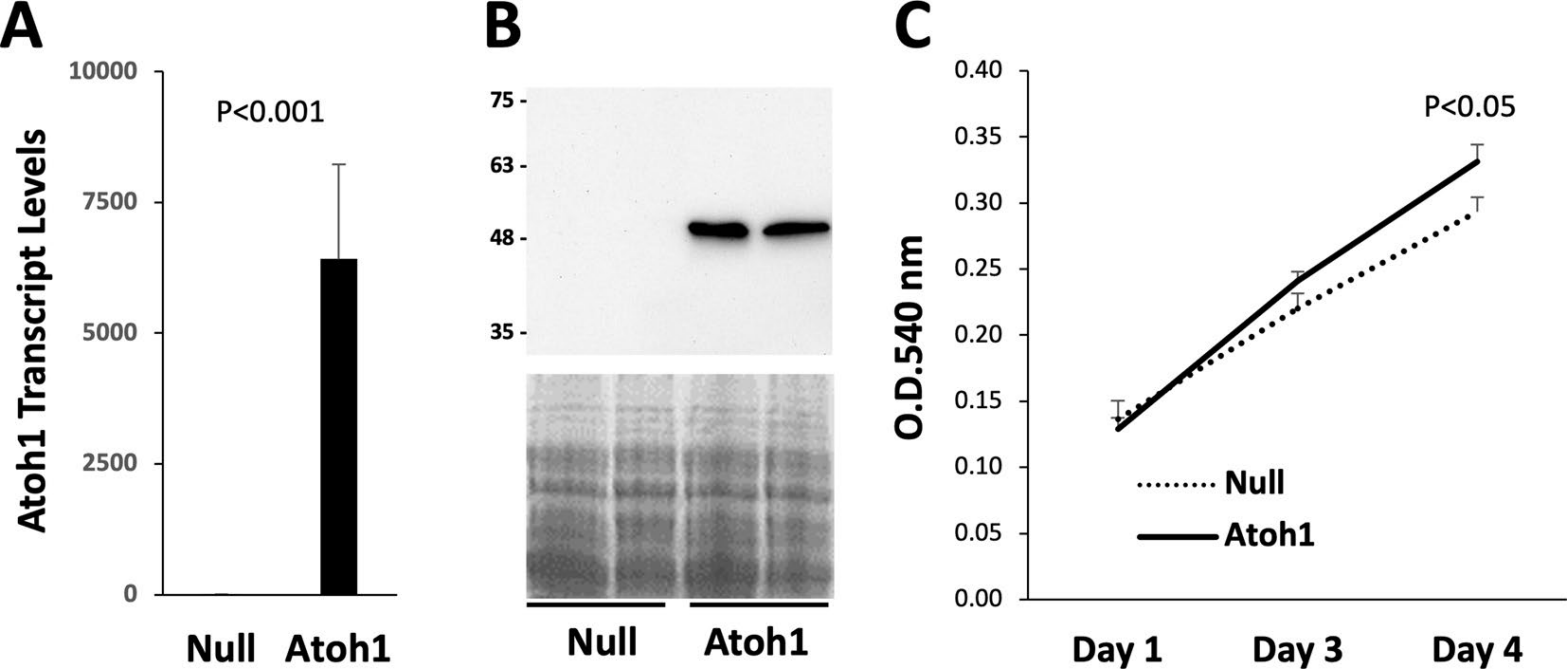
Ectopic expression of Atoh1 promotes DPSC proliferation. DPSCs treated with Adv-Null and Adv-Atoh1 were maintained in the growth medium. **A** Total RNA was isolated after 5 days for qPCR quantification of Atoh1 expression (*n* = 6). **B** Total proteins were extracted after 5 days for Western blotting analysis of Atoh1. **C** MTT cell proliferation assay was performed on day 1, 3, and 4 after viral vector treatments (*n* = 3)

Atoh1 is a developmentally regulated gene, and its postnatal expression is restricted to specific regions involved in the maintenance of sensory function and the epithelial cells of the gastrointestinal tract [6]. To test the possibility that Atoh1 may regulate the growth and differentiation of cells originated from the dental pulp, we used the replication-deficient adenoviral vector (Adv) [15] to achieve robust ectopic expression of Atoh1 in culture-expanded human DPSCs. Following treatments with Adv-Null (negative control) and Adv-Atoh1, qPCR analysis detected low-abundance Atoh1 transcripts with a threshold cycle greater than 32 in the Adv-Null-treated DPSCs. Adv-Atoh1-treated DPSCs exhibited an approximately 3,000-fold increase in the expression of Atoh1 compared to Adv-Null after 5 days (Fig. 1A). Western blotting reveals the abundance of Atoh1 protein in Adv-Atoh1-treated DPSCs but failed to detect Atoh1 protein in the control DPSCs (Fig. 1B). The high-level expression of Atoh1 led to significantly increased DPSC proliferation after 4 days in the growth medium (*P* < 0.05; Fig. 1C).

**Fig. 2:**
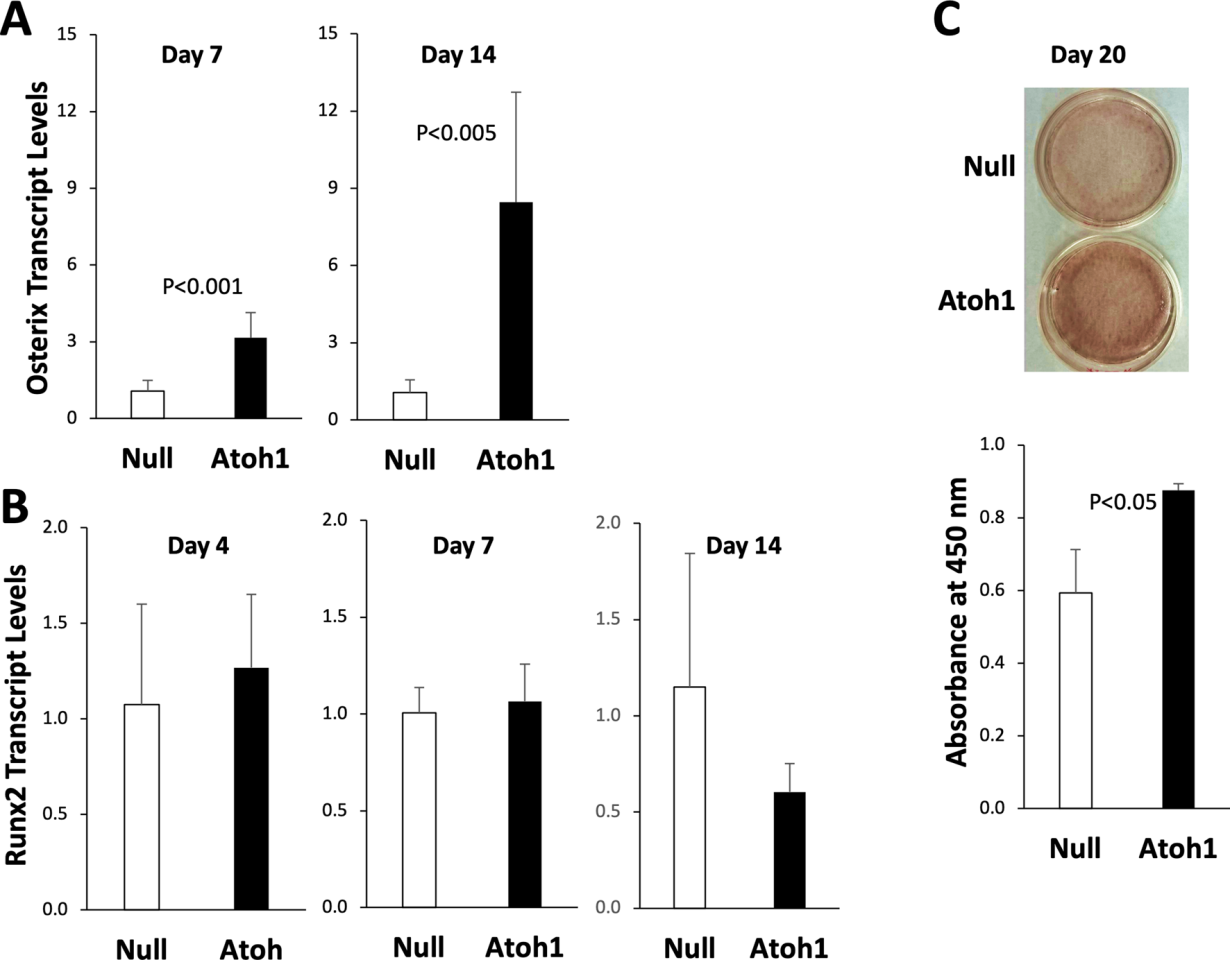
Ectopic expression of Atoh1 promotes odontogenic differentiation. DPSCs treated with Adv-Null and Adv-Atoh1 were maintained in the odontogenic medium. Total RNA was isolated for qPCR analysis. **A** Expression of Osterix on day 7 and 14 (*n* = 6). **B** Expression of Runx2 on day 4, 7, and 14 (*n* = 3 or 6). **C** DPSCs were fixed and stained with Alizarin Red on day 20 and photographed (top). Cell-bound Alizarin Red was extracted and quantified by light absorbance at 450 nm (bottom; *n* = 3)

We next examined the possibility that the overexpressed Atoh1 might affect the odontogenic cell fate of DPSCs maintained in the odontogenic medium. Osteogenic differentiation is primarily orchestrated by the two master transcription factors Runx2 and Osterix (also known as Sp7) [17]. Interestingly, Adv-Atoh1 significantly induced Osterix expression in a time-dependent manner: a 3-fold increase on day 7 (*P* < 0.001) and 8-fold increase on day 14 (*P* < 0.005) compared to Adv-Null (Fig. 2A). In contrast, Atoh1 did not exert any significant effect on Runx2 expression on day 4, 7, and 14 (Fig. 2B) presumably due to the abundance of Runx2 transcripts in DPSCs. Dlx5, another transcription factor involved in bone development [18], was also unaffected by Atoh1 (data not shown). DPSCs were maintained in the odontogenic medium for 20 days, following which cells were fixed and stained with Alizarin Red to reveal the extent of extracellular matrix mineralization. The staining shows that ectopically expressed Atoh1 significantly increased DPSC matrix mineralization (Fig. 2C; *P* < 0.05). Of note, the endogenous Atoh1 transcripts remained relatively unchanged throughout the early and late phase of DPSC odontogenesis (data not shown).

### Induction of collagen and dentin matrix proteins

**Fig. 3:**
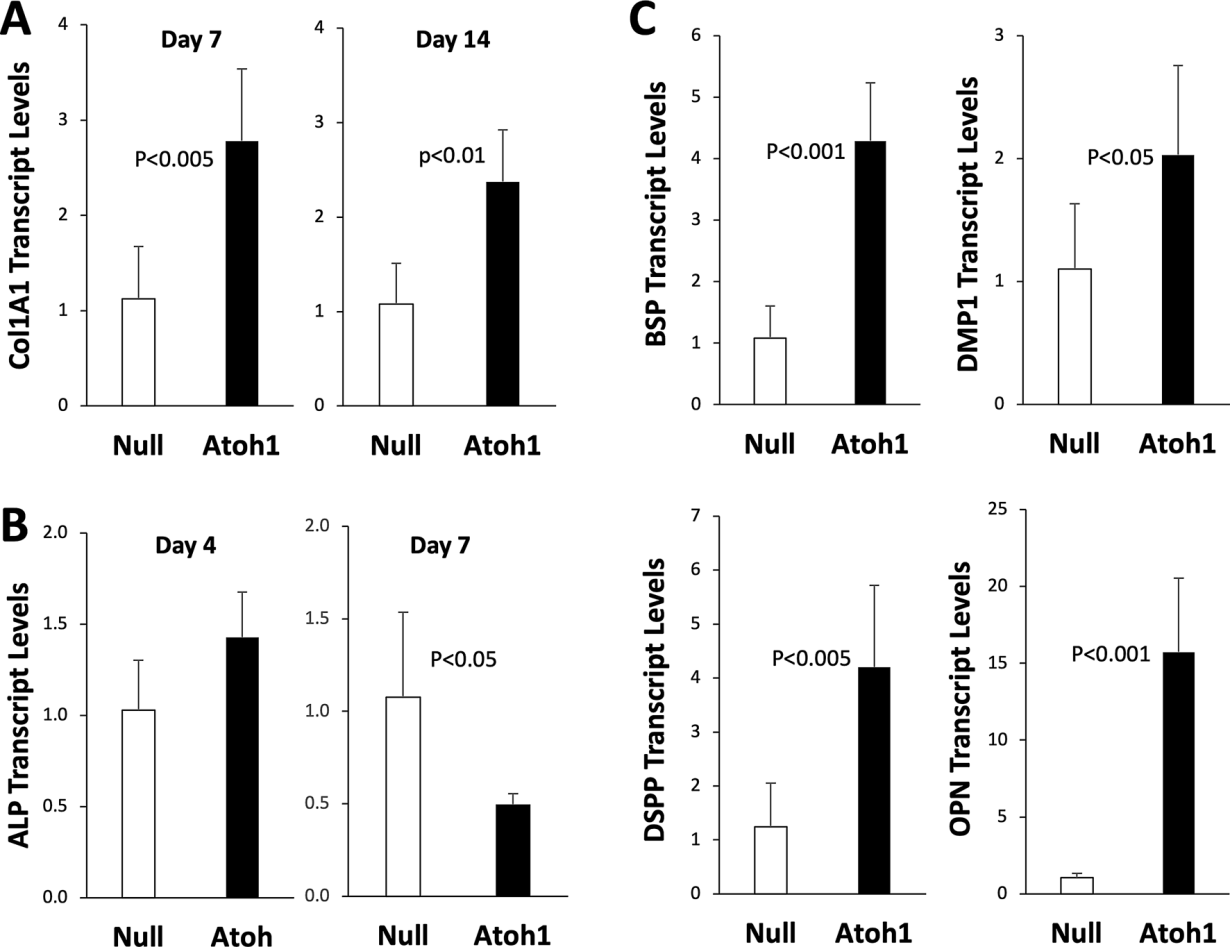
Atoh1-mediated induction of type I collagen and dentine matrix proteins. DPSCs treated with Adv-Null and Adv-Atoh1 were maintained in the odontogenic medium and total RNA was isolated for qPCR analysis. **A** Expression of Col1A1 on day 7 and 14 (*n* = 6). **B** Expression of ALP on day 4 and 7 (*n* = 3 or 6). **C** Expression of BSP, DMP1, DSPP, and OPN on day 7 (*n* = 6)

To further characterize Atoh1-mediated DPSC odontogenesis, we examined type I collagen gene expression, which is known to be transcriptionally activated by Osterix and Runx2 [17]. Figure 3A shows a significant induction of Col1A1 on day 7 (*P* < 0.005) and day 14 (*P* < 0.01). Alkaline phosphatase (ALP) is an ectoenzyme involved in collagen mineralization, which is a process of hydroxyapatite production within collagen fibrils [19]. Expression of ALP was slightly induced early on day 4, but significantly inhibited later on day 7 (Fig. 3B; *P* < 0.05), which was also confirmed by ALP enzyme activity assay (data not shown). Additional proteins involved in the mineralization of bone and dentin include the non-collagenous extracellular matrix proteins designated as small integrin-binding ligand, N-linked glycoproteins (SIBLINGs) [20]. Expression of several SIBLING genes were assessed by qPCR, including bone sialoprotein (BSP), dentin matrix protein 1 (DMP1), dentin sialophosphoprotein (DSPP), and Osteopontin (OPN). Figure 3C shows that Atoh1 significantly induced all SIBLING proteins on day 7: 4-fold induction of BSP (*P* < 0.001), 2-fold induction of DMP1 (*P* < 0.05), 4-fold induction of DSPP (*P* < 0.005), and most notably 15-fold induction of OPN (*P* < 0.001).

### Growth factor regulation and metabolic remodeling by Atoh1

**Fig. 4:**
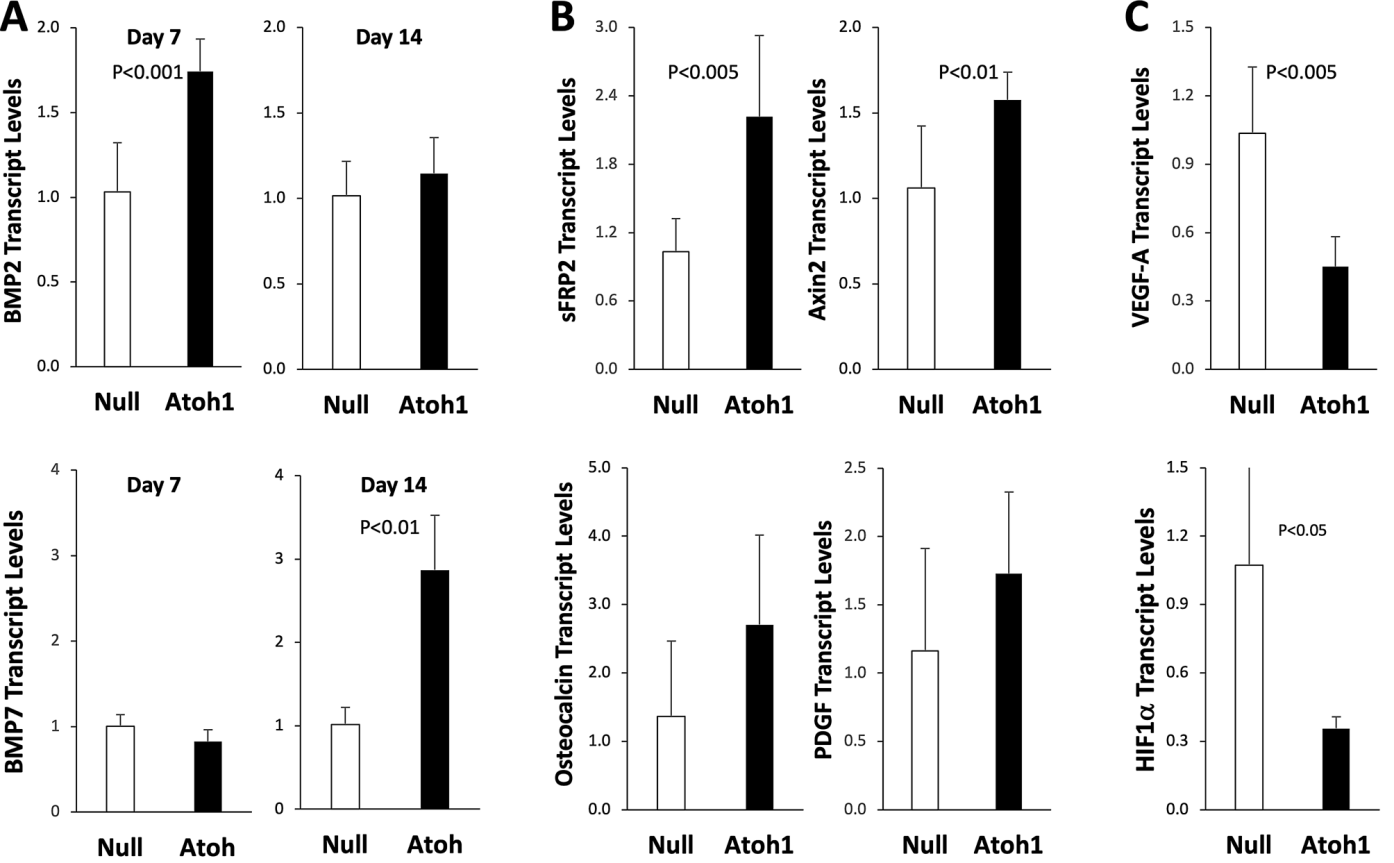
Growth factor regulation by Atoh1. DPSCs treated with Adv-Null and Adv-Atoh1 were maintained in the odontogenic medium. RNA was isolated for qPCR analysis. **A** Expression of BMP2 and BMP7 on day 7 and 14 (*n* = 3 or 6). **B** Expression of sFRP2, Axin2, PDGF, and Osteocalcin (*n* = 3 or 6) on day 7. **C** Expression of VEGF-A and HIF1α on day 7 (*n* = 3 or 6)

We previously demonstrated that DPSCs are a rich source of functionally diverse growth factors [14], some of which are involved in odontogenic regulation, such as BMPs and sFRP2 [21, 22]. Figure 4A shows that DPSCs overexpressing Atoh1 exhibited temporally regulated expression of BMP2 and BMP7 in that an early induction of BMP2 on day 7 (*P* < 0.001) was followed by a late induction of BMP7 on day 14 (*P* < 0.01). The result is thus consistent with the previous finding that Osterix is a downstream gene target of BMP2 [23]. Since Wnt signaling is known to promote DPSC differentiation into dentin-producing odontoblasts, we assessed Wnt activation using Axin2 expression as a readout [3, 24]. As predicted from the increased expression of sFRP2 (Fig. 4B; *P* < 0.005), Axin2 expression was significantly increased by Atoh1 (*P* < 0.01), suggesting an involvement of Wnt signaling in the action of Atoh1. In addition, Atoh1 slightly induced Osteocalcin and PDGF, but the effects were not statistically significant (Fig. 4B). Unexpectedly, Atoh1 significantly inhibited expression of VEGF-A (Fig. 4C; *P* < 0.005). The inhibition of VEGF appeared to be caused by downregulation of HIF1α because HIF1α was similarly inhibited by Atoh1 on day 7 (Fig. 4C; *P* < 0.05).

**Fig. 5:**
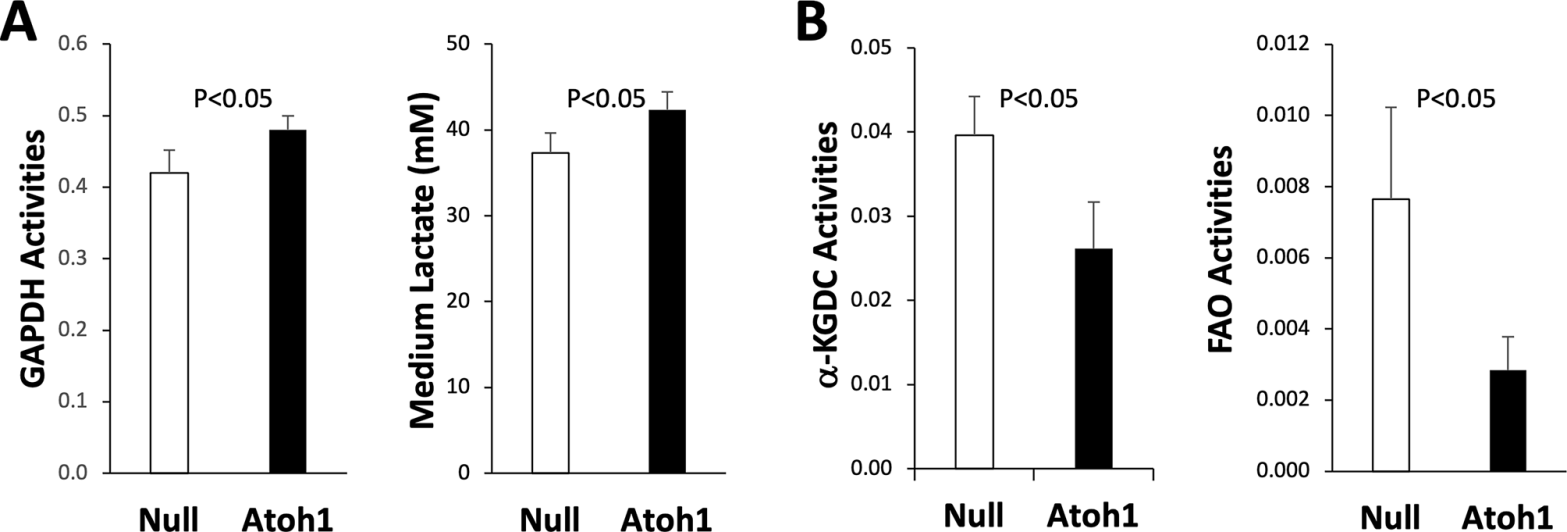
Metabolic remodeling by Atoh1. DPSCs treated with Adv-Null and Adv-Atoh1 were maintained in the odontogenic medium. Protein extracts and spent medium were prepared for enzyme activity and L-lactate assays on day 10. **A**: GAPDH activity assay using protein extracts and L-lactate assay using spent medium (*n* = 3). **B**: α-KGDC and FAO enzyme activity assays using protein extracts (*n* = 3)

Since growth factors can regulate nutrient metabolism through mTOR signaling [25], we went on to characterize energy metabolism in Atoh1-mediated DPSC odontogenesis. Glycolysis was assessed by measuring the activity of the house-keeping enzyme glyceraldehyde-3-phosphate dehydrogenase (GAPDH), which was significantly increased by Atoh1 (Fig. 5A; *P* < 0.05). Consistent with this finding, the level of L-lactate present in the spent medium was also significantly elevated (Fig. 5A; *P* < 0.05), indicating an increased glycolytic flux. Mitochondrial energy metabolism was assessed by measuring the activities of the Krebs Cycle enzyme α-ketoglutarate dehydrogenase complex (α-KGDC) and fatty acid oxidation (FAO). Both α-KGDC (*P* < 0.05) and FAO (*P* < 0.05) enzyme activities were significantly reduced by Atoh1 (Fig. 5B), suggesting a downregulation of aerobic nutrient metabolism mediated by Atoh1. Taken together, the current study unravels a novel odontogenic mechanism mediated by ectopic expression of Atoh1 in DPSCs and the mechanism is associated with metabolic remodeling.

## Discussion

Developmentally regulated transcription factors acting in concert form the gene regulatory networks that vary in different cellular contexts and represent the major drivers in cell fate determination. Some transcription factors possess the extraordinary ability to reprogram cell fate, the most notable finding being the Yamanaka (OSKM) factors, which upon ectopic expression can convert somatic cells to pluripotent cells [26]. bHLH transcription factors are well known for their roles in cell lineage specification and differentiation. Although Atoh1 is known to act as a proneural transcription factor essential for auditory hair cell regeneration in the sensory epithelium of mammalian auditory and vestibular tissues [5, 6], it has also been found to drive Merkel cell production in the epidermis upon ectopic expression [27]. The current study uncovered a remarkable ability of ectopically expressed Atoh1 in promoting DPSCs toward the odontogenic cell fate, although this cell fate determination mechanism is unlikely to be physiological. Given the similarities between DPSCs and mesenchymal stem cells (MSCs), we speculate that Atoh1 may also possess the ability to drive MSCs toward the osteogenic cell fate upon ectopic expression in MSCs.

The mechanism underlying the observed pro-odontogenic function of Atoh1 remains speculative at present. Atoh1, being a member of the bHLH family of dimeric transcription factors, activates or represses gene promoters harboring the E-box motif through differential interactions with other bHLH members [5, 6]. A well-established example of gene regulation by bHLH dimers is that mediated by MyoD partnering with E2A in myogenesis, which can be antagonized by MyoD-Id1 dimer [28]. Indeed, it has been found that Atoh1 can transiently activate gene expression in collaboration with the bHLH member TCF3/E47, but this activity is greatly attenuated when Atoh1 partners with another bHLH member HES1 [29]. Additional bHLH members implicated in bone development and skeletal maintenance include Hand1, Twist1 and Twist2 [27, 30, 31]. Since DPSCs do not appreciably express Atoh1, ectopic expression of Atoh1 in DPSCs may enable Atoh1 to cooperate with Hand1 and Twist1/2, thus altering the bHLH regulatory landscape and leading to the observed odontogenic phenotype. Given that both Hand1 and Twist1/2 can function as pioneer factors [32, 33], the presence of abundant Atoh1 in DPSC nuclei can help initiate opening of the closed chromatin through dimerization with Hand1 or Twist1/2, thus increasing transcriptional output on those genes encoding odontogenic signaling molecules, transcription factors, and dentin matrix proteins. The interaction between Atoh1 and the pioneer factor POU4F3 in driving hair cell and Merkel cell differentiation has indeed been demonstrated in which Atoh1 stimulates the expression of POU4F3 to facilitate unpacking of lineage-specific chromatin structures [34].

Our study shows that Atoh1-mediated DPSC odontogenesis is associated with induction of key signaling pathways orchestrated by BMP2, BMP7, and the Wnt regulator sFRP2. The importance of Wnt signaling in odontogenesis is underlined by the finding that activation of Wnt signaling through inhibition of GSK3 has been shown to promote natural tooth repair [35]. Indeed, Atoh1 has been found to cooperate with Wnt/β-catenin to achieve hair cell regeneration in postnatal mouse cochleae [36]. BMPs provide major signaling cues important for osteogenesis and odontogenesis and have found their applications in the orthopedic field [37]. BMP2 is known for its roles in tooth and bone regeneration and can broadly induce osteogenic transcription factors such as Osterix, Runx2, and Dlx5 [23, 38]. Interestingly, Atoh1-mediated induction of BMP2 and BMP7 appears temporally regulated in that transient induction of BMP2 precedes that of BMP7 during DPSC odontogenesis, suggesting that members of the BMP gene family may be differentially involved in the early and late phases of DPSC odontogenesis. To our surprise, we observe that Atoh1-mediated DPSC odontogenesis is associated with downregulation of the HIF1α/VEGF-A axis. Since HIF1α induces VEGF and has been shown to promote bone formation by coupling osteogenesis and angiogenesis [39], Atoh1-mediated odontogenesis may require additional signaling cues such as hypoxia to achieve fully functional and mature odontoblasts.

Runx2 and Osterix are two master transcription factors known to interact and coordinately regulate the expression of bone/tooth-specific genes [40]. Runx2 expression typically occurs at an early stage of osteogenic differentiation, which is followed by induction of Osterix during the bone/tooth maturation phase. Osterix is required for the proliferation and differentiation of ameloblasts and odontoblasts [41]. Interestingly, we found that ectopically expressed Atoh1 induced Osterix, but not Runx2 or Dlx5. This can perhaps be explained by our observation that both Runx2 and Dlx5 transcripts are constitutively present in DPSCs and are at least 100 times more abundant than Osterix transcripts, suggesting that the DPSCs used in the current study, which are maintained in Advanced DMEM, may have been primed for odontogenesis. The abundance of Runx2 and Dlx5 proteins present in the DPSCs may thus be adequate for functional and physical interaction with Osterix, which is deemed necessary for efficient induction of bone/tooth-specific genes [40], as also evident from Atoh1-mediated upregulation of collagen and non-collagenous matrix proteins.

A previous characterization of the Atoh1-mediated targetome reveals that Atoh1 can regulate over 600 genes including some house-keeping genes and those involved in cell metabolism [9]. Our data show that Atoh1 promoted DPSC odontogenesis through regulation of multiples genes encoding growth factors, transcription factors, SIBLINGs, and those involved in energy metabolism. Specifically, Atoh1-mediated DPSC odontogenesis is associated with metabolic remodeling marked by increased glycolytic flux and decreased activities of Krebs Cycle and FAO enzymes. In the same vein, we note that deletion of hexokinase 2 in the intestinal epithelium was found to cause a decreased glycolytic flux along with increased Atoh1 expression [42]. Although the significance of the possible association between Atoh1 expression and glucose metabolism is presently unclear, we note that Atoh1 can function as an oncogene in medulloblastomas or a tumor suppressor gene in adenomatous polyposis carcinoma [6]. Given that oncogenesis is intricately influenced by metabolic remodeling, a key cellular function of Atoh1 may involve metabolic regulation in cell growth and differentiation.

## Conclusions

In summary, although Atoh1 is known for its roles in neural and epithelial development, the current study demonstrates an odontogenic mechanism mediated by robust ectopic expression of Atoh1 in DPSCs. The discovery may have implications for developing innovative gene-based strategies for bone and tooth repair.

## Electronic supplementary material

Below is the link to the electronic supplementary material.

Supplementary Material 1

## Data Availability

The datasets used and/or analyzed during the current study are available from the corresponding author on reasonable request.

## Abbreviations

Atoh1: Atonal homolog 1
bHLH: Basic helix-loop-helix
DPSC: Dental pulp stem cell
BMP: Bone morphogenetic protein
SIBLINGs: Small integrin-binding ligand, N-linked glycoproteins
DMSO: Dimethyl sulfoxide
B2M: β2 microglobulin
SDS-PAGE: Sodium dodecyl-sulfate polyacrylamide gel electrophoresis
GAPDH: Glyceraldehyde-3-phosphate dehydrogenase
TRAP: Tartrate-resistant acid phosphatase
α-KGDC: α-ketoglutarate dehydrogenase complex
FAO: Fatty acid oxidation
MSC: Mesenchymal stem cell
