# Supplementary figures and images for: The proneural transcription factor Atoh1 promotes odontogenic differentiation in human dental pulp stem cells (DPSCs)
Source: BMC Mol Cell Biol. 2025 Jan 20;26:5. doi: 10.1186/s12860-025-00530-2 (PMC11744864; doi:10.1186/s12860-025-00530-2)

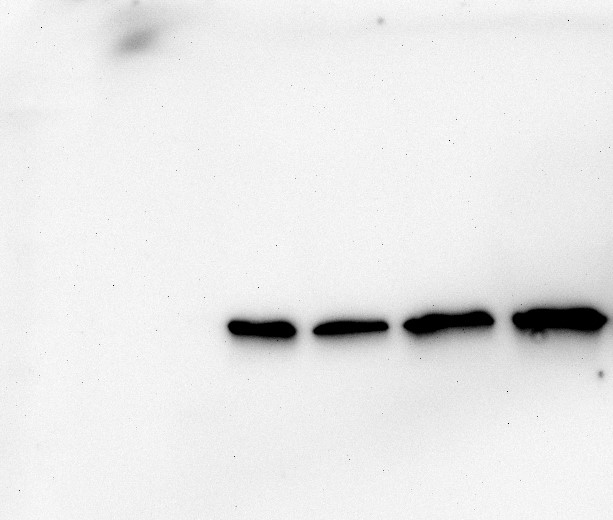

Supplement: Supplementary file 1 — Supplementary Material 1 [file 12860_2025_530_MOESM1_ESM.jpg]
